# Retraction for Megahed et al., “Complete Genome Sequence of White Spot Syndrome Virus Isolated from Indian White Prawn (*Fenneropenaeus indicus*) in Egypt”

**DOI:** 10.1128/MRA.01267-19

**Published:** 2019-10-31

**Authors:** Mohamed E. Megahed, Siddhartha Kanrar, Arun K. Dhar

**Affiliations:** a National Institute of Oceanography and Fisheries (NIOF), Gulfs of Suez & Aqaba’s Branch, Attaka, Suez, Egypt; b Aquaculture Pathology Laboratory, School of Animal and Comparative Biomedical Sciences, The University of Arizona, Tucson, Arizona, USA

## RETRACTION

Volume 8, no. 1, e01508-18, 2019, https://doi.org/10.1128/MRA.01508-18. We retract this article. The sequence was submitted to the NCBI GenBank (accession no. KR083866) on 13 April 2015 by the primary author, Mohamed E. Megahed, National Institute of Oceanography and Fisheries (NIOF), Suez, Egypt. Unfortunately Dr. Megahed has been instructed by NIOF, Suez, Egypt, not to share the raw sequence data under the Law No. 82/2002 and via a declaration of the Deputy Minister of Agriculture & Land Reclamation. Due to this inability to share the raw sequence data, we are retracting the article.

